# Tissue Quality Assessment Using a Novel Direct Elasticity Assessment Device (The E-Finger): A Cadaveric Study of Prostatectomy Dissection

**DOI:** 10.1371/journal.pone.0112872

**Published:** 2014-11-10

**Authors:** Daniel W. Good, Ashfaq Khan, Steven Hammer, Paul Scanlan, Wenmiao Shu, Simon Phipps, Simon H. Parson, Grant D. Stewart, Robert Reuben, S. Alan McNeill

**Affiliations:** 1 Edinburgh Urological Cancer Group, University of Edinburgh, Western General Hospital, Edinburgh, EH2 4XU, United Kingdom; 2 School of Engineering and Physical Sciences, Heriot Watt University, Edinburgh, United Kingdom; 3 Department of Anatomy, University of Edinburgh, Edinburgh, United Kingdom; 4 Department of Urology, Western General Hospital, NHS Lothian, Edinburgh, United Kingdom; National Health Research Institutes, Taiwan

## Abstract

**Introduction:**

Minimally invasive radical prostatectomy (RP) (robotic and laparoscopic), have brought improvements in the outcomes of RP due to improved views and increased degrees of freedom of surgical devices. Robotic and laparoscopic surgeries do not incorporate haptic feedback, which may result in complications secondary to inadequate tissue dissection (causing positive surgical margins, rhabdosphincter damage, etc). We developed a micro-engineered device (6 mm^2^ sized) [E-finger]) capable of quantitative elasticity assessment, with amplitude ratio, mean ratio and phase lag representing this. The aim was to assess the utility of the device in differentiating peri-prostatic tissue types in order to guide prostate dissection.

**Material and Methods:**

Two embalmed and 2 fresh frozen cadavers were used in the study. Baseline elasticity values were assessed in bladder, prostate and rhabdosphincter of pre-dissected embalmed cadavers using the micro-engineered device. A measurement grid was created to span from the bladder, across the prostate and onto the rhabdosphincter of fresh frozen cadavers to enable a systematic quantitative elasticity assessment of the entire area by 2 independent assessors. Tissue was sectioned along each row of elasticity measurement points, and stained with haematoxylin and eosin (H&E). Image analysis was performed with Image Pro Premier to determine the histology at each measurement point.

**Results:**

Statistically significant differences in elasticity were identified between bladder, prostate and sphincter in both embalmed and fresh frozen cadavers (p = <0.001). Intra-class correlation (ICC) reliability tests showed good reliability (average ICC = 0.851). Sensitivity and specificity for tissue identification was 77% and 70% respectively to a resolution of 6 mm^2^.

**Conclusions:**

This cadaveric study has evaluated the ability of our elasticity assessment device to differentiate bladder, prostate and rhabdosphincter to a resolution of 6 mm^2^. The results provide useful data for which to continue to examine the use of elasticity assessment devices for tissue quality assessment with the aim of giving haptic feedback to surgeons performing complex surgery.

## Introduction

Prostate cancer is the fourth most common cancer and the most common non-dermatologic cancer in men in the UK [1]. Worldwide it accounted for approximately 14% of all new male cancers diagnosed in 2008 [2]. Radical Prostatectomy (RP) is a treatment option for localised and locally advanced prostate cancer [3]. Minimally invasive RP (robotic assisted radical prostatectomy - RARP and laparoscopic radical prostatectomy - LRP), have brought improvements in the pentafecta outcomes of RP [4]; specifically, early continence [5], improved potency [6] and reduced positive surgical margins (PSM) [7] due to improved views and increased degrees of freedom of surgical devices. RARP and LRP, however, do not enable haptic feedback [8] which ultimately may result in oncological and functional complications from surgery.

Incision into the prostate or stripping of the capsule near a tumour during radical prostatectomy may result in a PSM, which in turn is an independent predictor of biochemical recurrence for prostate cancer [9] - apical margins are particularly common with LRP [9]. There is evidence that damage to the rhabdosphincter during apical dissection leads to worse recovery of urinary incontinence [10], [11]. There is much interest in the use of haptic feedback devices for tissue assessment intra-operatively [8] as it is hoped that this will lead to improved outcomes from surgery. Elasticity assessment devices have been used in the detection of prostate cancer but the extension into detailed tissue assessment has been so far lacking [12].

### Elasticity and Dynamic Instrumented Palpation

Elasticity is a measure of the specific stiffness of an object [12]. Biological tissues such as the prostate do not behave in a purely linear elastic manner instead they behave in a viscoelastic manner – related to the proportions of viscous and elastic tissue that make up the specific tissue. Current prostate elastic theory [13] states that the epithelial tissue predominantly composed of acini (water filled glands) act in a viscous manner whereas the stromal (predominantly elastic smooth muscle) component acts in an elastic manner. The bladder and sphincter complex areas are histologically distinct from the prostate and as such identifying differences in elasticity should be possible.

To our knowledge there has been no work done on the difference in elasticity between bladder, prostate and rhabdosphincter (sphincter). Applying the current viscoelastic model to other tissues we would expect the bladder and rhabdosphincter (predominantly composed of muscle) to behave more elastically (have lower amplitude ratio (AR), mean ratio (MR)) in comparison to prostatic tissue.

Dynamic instrumented palpation (DIP) is a novel concept for the elastic assessment of biological tissues. It involves the use of a device (E-finger) to produce an oscillatory indentation displacement to a tissue. The resulting force being recorded (time dependent). The force response is sinusoidal and therefore the phase difference between the load and displacement and amplitude ratio can be determined. The dynamic elasticity components: amplitude ratio is thought to be related to the elastic component and the phase lag (PL) the viscous component of the tissue [14]. The mean ratio, a quasi-static elasticity parameter, is hypothesized to relate to a combination of both components [15].

A cadaveric model was used to investigate the use of elasticity in tissue assessment using a novel direct elasticity assessment device (E-finger). The cadaveric model was used to determine if elasticity measurements made with E-finger can be used to differentiate the organs and organ tissues of the lower urinary tract that in turn may be used to assess tissue during prostatectomy dissection.

## Materials and Methods

Local ethical approval was obtained from the Anatomy Department, University of Edinburgh for all the research conducted and all tests were performed within the Department of Anatomy. The cadavers used were registered for research use and donated by the patients/next of kin by written informed consent (http://www.anatomy.mvm.ed.ac.uk/bequests/). In total four cadavers were utilised for the study, two were embalmed cadavers and two were fresh frozen (thawed) cadavers.

**Figure 1:**
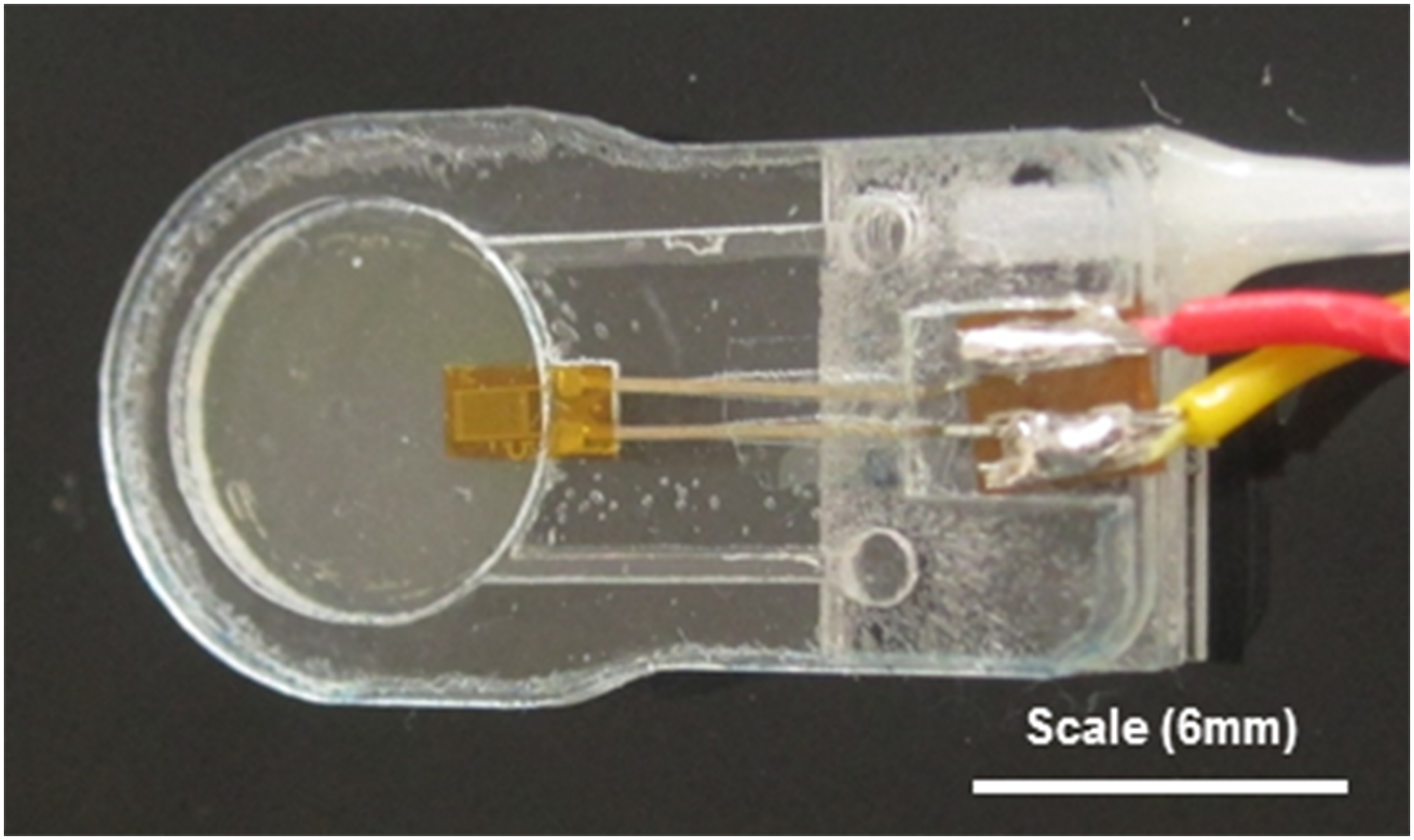
Picture of the prototype E-finger device.

The E-finger device (Figure 1) [15] has a dynamically actuated membrane which is moved by a pulsatile compressed air flow and is pulsed at a specific frequency. This is supported by an outer casing in order to secure the membrane in place. A strain gauge is mounted to the membrane to measure its deflection response. The device was pressurised cyclically at a rate of between 1 Hz and 15 Hz with a peak air pressure of 0.5 bar. The device, in this study, was held by the examiner on the tip of their index finger underneath a latex glove. The phase angle and amplitude and mean ratio between the applied pressure and the membrane strain gauge signal are used to obtain the tissue dynamic modulus (expressed as amplitude ratio (AR), mean ratio (MR) and phase lag (PL)). The device is used by pressing it against the surface of the tissue being examined with constant static pressure, whilst dynamically pulsing the membrane.

The study was designed to test the null hypothesis that there was no statistically significant difference between the elasticity values of bladder, prostate and the rhabdosphincter complex. Two separate studies were conducted; initially (study 1) using 2 embalmed cadavers and then progressing to use 2 fresh frozen cadavers (study 2).

Study 1: Areas on 2 pre-dissected embalmed cadavers were selected which were identified visually as bladder, prostate or rhabdosphincter complex on both the anterior and lateral surfaces. This study sought to determine if different elasticity values were demonstrable before moving onto study 2.

**Figure 2:**
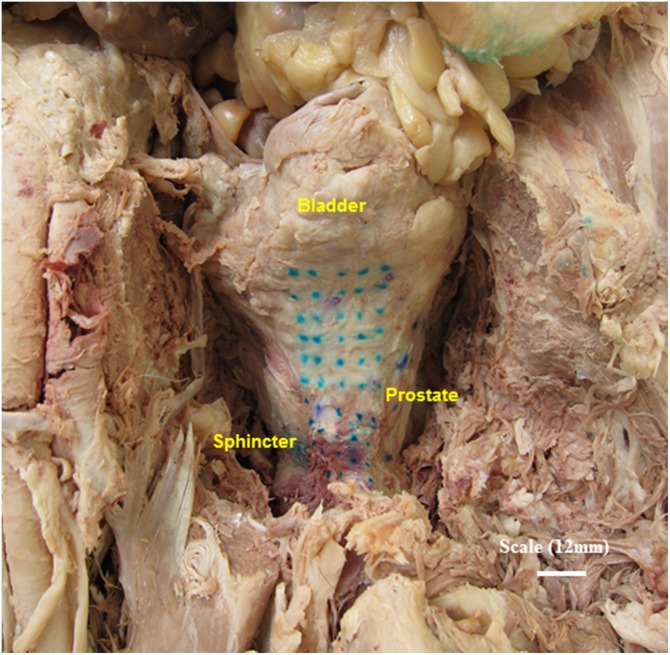
A Picture showing the grid (Markings) for the assessment of elasticity on an embalmed cadaver.

Study 2: A grid (Figure 2) was created on the anterior surface of 2 non-dissected fresh frozen (thawed) cadavers. The grid spanned a large area such as to cover areas of the bladder, prostate and sphincter using visual cues as a guide. Each point on the grid varied by the dimensions of the probe size (6 mm), such that a systematic assessment of the entire area was conducted (Figure 2). Ink dots and then pins were placed on the measurement areas. Two independent assessors (DG, AK) assessed each area of the grid using the E-finger attached to the index finger of their hands.

**Figure 3:**
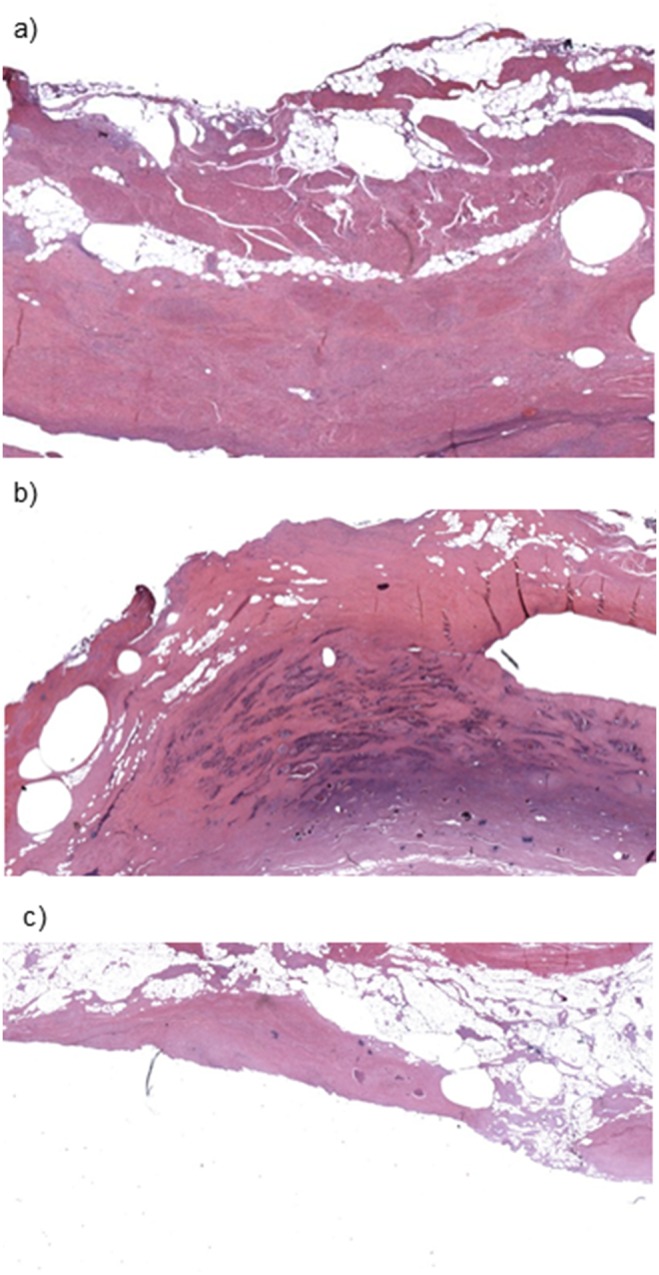
Histological image (H&E staining) x10 magnification showing bladder smooth muscle tissue.

Each horizontal line of measurement points from superior to inferior aspects (Figure 2) were then cross-sectioned at 6 mm intervals, fixed and wax embedded. Each horizontal cross-section was then further cut so that 5-micron thick cross-sections were available and haematoxylin and eosin (H&E) staining was performed. These cross-sectional H&E slides were then analysed with image analysis software (Image Pro Premier, Media Cybernetics, UK). Image analysis was used to calculate the percentage of each tissue type (bladder, prostate, and sphincter) so that areas with the greatest proportion of each tissue type were labelled as either bladder, prostate or sphincter (figure 3). Each of these areas corresponded to the column of measurement assessed by the E-finger probe. This method allowed the greatest locational accuracy for correlation of elasticity to underlying histology.

After assessments had been made the elasticity values and histological data were entered into a Microsoft Excel spreadsheet (Chicago, USA) and differences between various tissue types calculated.

### Statistical Analysis

Data was analyzed using SPSS version 19 (Chicago, USA) and Salford Systems CART (San Diego, USA). Non-parametric data was analysed using the Mann Whitney U and Kruskal-Wallis tests. The null hypothesis was that there was no statistical difference in the elasticity values between different tissues types. P values were set at <0.05. Univariate logistic regression was used to identify which elasticity outcome measures at a specific frequency were significant predictors of tissue type (bladder, prostate or sphincter mechanism). The variables identified as significant on univariate regression were modelled together, in a multivariate logistic regression model, to identify independent predictors of tissue type. On the combined data from both fresh frozen cadavers, Classification and Regression Tree (CART) analysis was performed. Here the software created a model using only the devices’ elasticity data and its accuracy for detecting peri-prostatic tissue was assessed.

## Results

### Study 1

**Figure 4:**
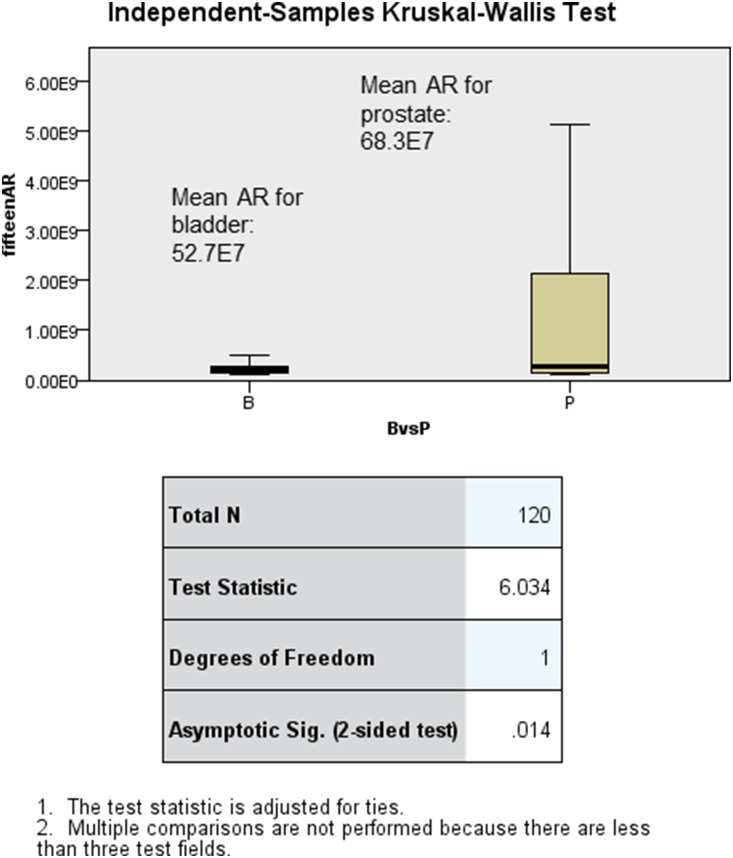
Boxplots of 15 Hz-AR for prostate and bladder tissue, showing a statistically significant difference (p = 0.014).

**Figure 5:**
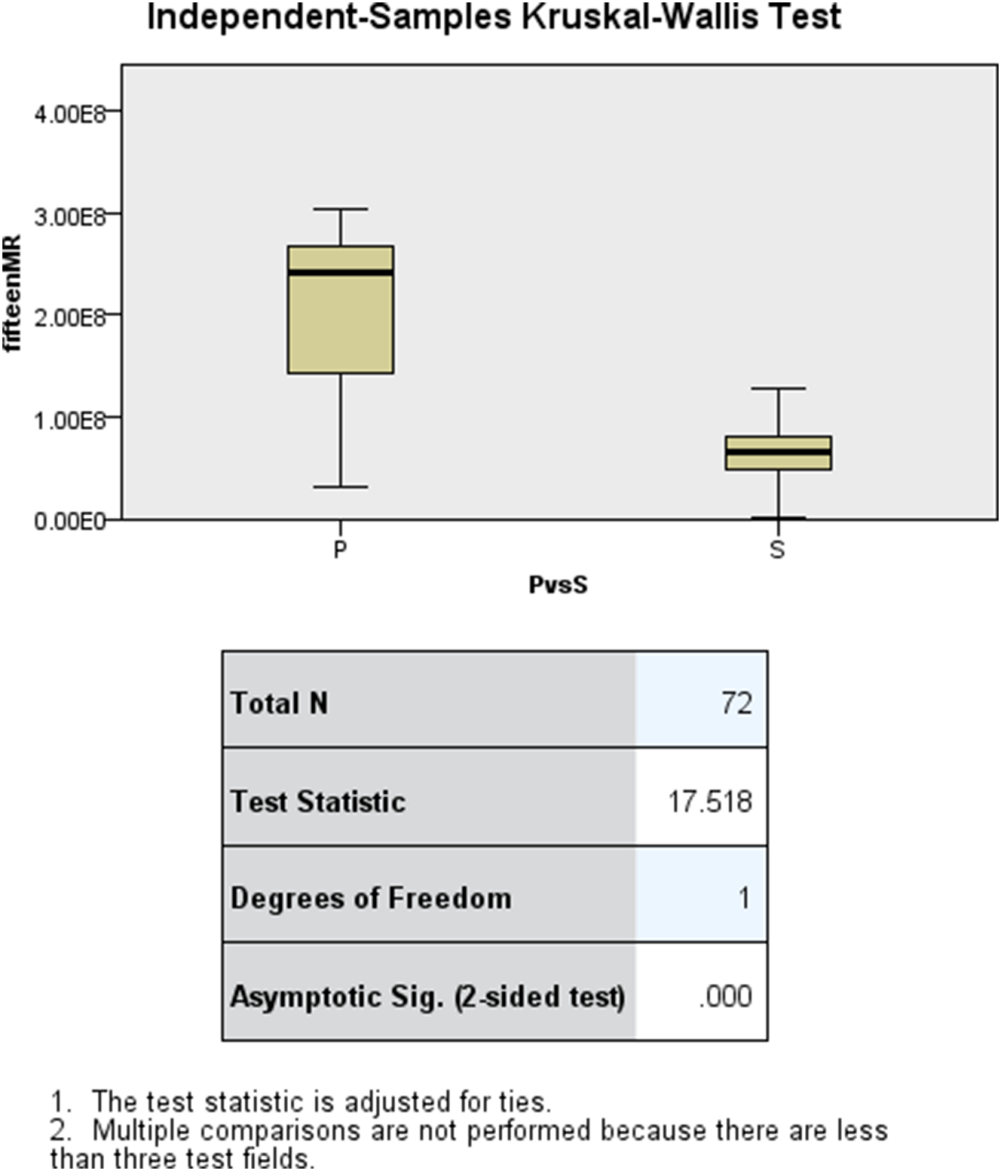
Boxplots of 15 Hz-MR for prostate and sphincter tissue, showing a statistically significant difference (p = <0.001).

In total this study yielded 192 elasticity values from assessments on the anterior (Figure 2) and both lateral surfaces of the two pre-dissected embalmed cadavers. 120 measurements were used to detect differences between bladder and prostate areas and 72 for prostate and sphincter areas. Univariate logistic regression analysis showed 5 Hz-AR, 5 Hz-MR, 10 Hz-AR, 15 Hz-MR and 15 Hz-AR being significant predictors of prostate from bladder tissue. On combining these into a multivariate logistic regression model, 15 Hz-AR was a significant independent predictor of tissue type (p = 0.01). Figure 4 shows the boxplots of 15 Hz-AR for bladder and prostate tissues and shows that the differences between these are statistically significantly (p = 0.014). Similarly, on multivariate regression analysis, 15 Hz-MR was an independent predictor of tissue type (p = 0.011). Five Hz-MR, 10 Hz-MR, 10 Hz-PL and 15 Hz-MR were significant predictors on univariate analysis, for identifying prostate from sphincter tissue types. Figure 5 shows the boxplots of 15 Hz-MR for prostate and sphincter tissues and shows that these are statistically significantly different (p = <0.001).

### Study 2

In this study two non-dissected fresh frozen cadavers, which were thawed for the assessments, were used. In total this study yielded: cadaver 1, 35 different measurement points; in cadaver 2, 60 measurements owing to the larger size of the prostate (60 g prostate weight vs 30 g).

Univariate logistic regression analysis for cadaver 1 revealed that 1 Hz-AR, 5 Hz-MR, 10 Hz-MR and 15 Hz-MR were significant predictors of tissue type. The multivariate logistic regression model shows that 1 Hz-AR was the best predictor, although this was not statistically significant (p = 0.062). Kruskal-Wallis analysis showed that there was a statistically significant difference between bladder, prostate and sphincter, with prostate having a higher AR than bladder and sphincter (p = <0.001).

Similarly for cadaver 2, univariate logistic regression analysis yielded 5 Hz-AR, 10 Hz-AR, 1 Hz-AR and 10 Hz-MR being significant predictors of tissue type. The multivariate logistic regression model shows that 10 Hz-AR was the most likely predictor, however, this missed statistical significance (p = 0.053). Kruskal-Wallis analysis showed that there was a statistically significant difference between bladder, prostate and sphincter, with prostate having a higher AR than bladder and sphincter (p = <0.005).

To assess if the results from individual cadavers were generalizable to other cadavers, data from both cadaver 1 and 2 (both fresh-frozen) were combined and similar analysis performed.

Univariate logistic regression revealed that 1 Hz-AR, 5 Hz-AR, 10 Hz-MR, 10 Hz-AR, 15 Hz-MR and 15 Hz-AR were significant predictors of tissue type (bladder vs prostate). On multivariate analysis 5 Hz-AR was the strongest independent predictor of tissue type (p = 0.024). Mann-Whitney U test showed that there was a statistically significantly higher AR for prostate than bladder (p = <0.001). For prostate vs sphincter, 5 Hz-AR (p = 0.015) on multivariate regression was an independent predictor of tissue type. Mann Whitney U test revealed a statistically significantly lower AR for sphincter compared to prostate tissue (p = 0.001).

To assess inter-rater reliability for the test, intra-class correlation coefficient (ICC) analysis was performed for assessor 1 and assessor 2 (study 2). This yielded an average measure coefficient of 0.731 (95% CI: 0.519–0.849).

**Figure 6:**
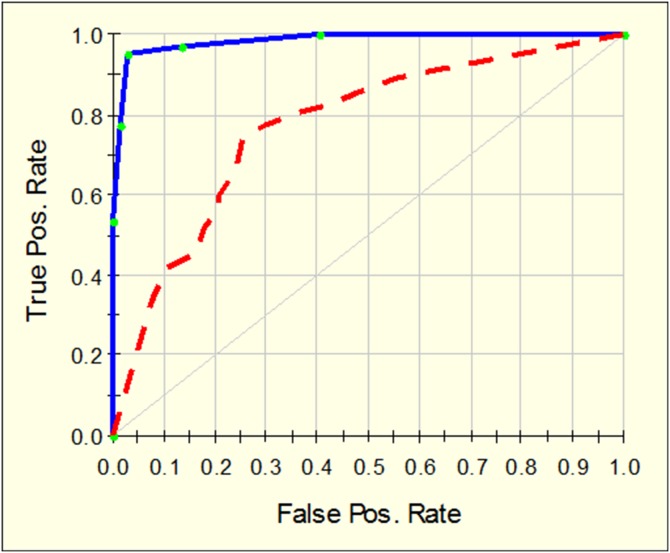
ROC for 15 node CART model on learn (blue line) and test (red line).

CART analysis identified a model containing 15 nodes from purely mechanical data created from the device. Figure 6 shows the area under the curve (ROC) for the learning of the model (0.98) and also for the testing of the model (0.76). The model revealed a sensitivity and specificity of 77% and 70% respectively for the identification of prostatic tissue within the entire dataset of study 2.

## Discussion

This study has demonstrated that there are clear and quantifiable differences in elasticity between peri-prostatic tissue types and that these are differences are measurable using a micro-scale device capable of being used in-vivo. This study used a systematic scanning technique and was capable of tissue identification with a sensitivity of 77%. Furthermore these differences were generalizable across 2 different fresh frozen cadavers, a finding that has important potential clinical implications should these results be replicated in vivo. The fact that these differences were measurable even in embalmed tissues, a process which makes human tissue far stiffer, suggests that measurable differences will also be evident in live human tissue.

The testing of a micro-scale, direct quantitative elasticity assessment device capable of deployment in-vivo, which has so far not been achieved due to the underlying engineering challenges of micro-scaling these devices, is a significant strength of this study [12]. In addition this is the first study looking at identification of quantitative differences in elasticity between peri-prostatic tissue types that has potential clinical applications for tissue quality assessment and haptic feedback. The rigorous methodology used to strengthen the locational accuracy for correlation of the elasticity measurements with underlying histology means that we can be confident that these differences are real and not simply due to sampling error.

We do recognise that our study has some weaknesses, which include that measurements in cadaveric tissue may not be transferable to live human tissue. There are real differences between live tissue and in particular embalmed cadavers. However the fact that we were able to use fresh frozen (thawed) cadavers, which are far more like live human tissue as there is no tissue fixative, and still identify these differences suggests that similar differences will be measurable in vivo. Another weakness in the study is the small number of cadavers used (n = 4–2 embalmed and 2 fresh frozen) which risk the study being underpowered. However, the large numbers of measurement points taken from each cadaver reduces the risk of statistical errors. Furthermore, significant differences were also detected using regression analysis, which suggests that the study was adequately powered to allow us to confidently reject our null hypothesis.

The study (study 2) identified that prostate tissue had a higher amplitude ratio and mean ratio (greater stiffness) than bladder and rhabdosphincter (p = <0.001) and (p = 0.001) respectively. This finding is consistent with our hypothesis and we believe that it is due to the underlying differences in histology. The prostate being predominantly made up of both viscous (acini) and elastic (stromal) areas (behaves visco-elastically) [14], [16], which differentiates it from bladder (smooth muscle) and rhabdosphincter which lack this viscous element. We do acknowledge that the accuracy of detection was not 100% and this is likely due to areas where there was a mixture of tissue types in the area of assessment rather than purely one type. There are no other studies in the published literature, to the best of our knowledge, looking at the differences in peri-prostatic tissues, despite there being a large evidence base when confined to the prostate. This is likely due to the engineering challenges of micro-scaling which means performing a study such as ours is not possible inside a cadaver or human.

Another interesting observation from our study is that the frequency of membrane activation was very low (range 1–15 Hz), and on the fresh frozen cadavers (study 2) 5 Hz detected differences most effectively. This contrasts with the findings of many other groups who used large mechanical indenters at relatively higher frequencies for identification of prostate disease within prostates [16]–[18]. The decision to use a lower frequency came about from previous work from our group where we showed using lower frequency was better able to differentiate benign from malignant prostate tissue [14].

This study also revealed the potential advantages of dynamic instrumented palpation (DIP) over the static assessment techniques used by other groups. This static measurement was represented in our device output by the quasi-static outcome of mean ratio. In our study AR was a better differentiator of tissue type than MR, this was consistent among both fresh frozen cadavers, among both assessors and is evidenced by the multivariate regression analysis for study 1 and 2 combined. MR was not an independent predictor of tissue type with fresh frozen cadavers. The evidence presented here suggests that for peri-prostatic tissues, DIP is a technique which can result in better differentiation of tissues than a static measure as more information is gained when tissues are assessed continuously over a time period. The resulting differences are likely to be greater when there is a change in the tissue-type being assessed (prostate from bladder or sphincter or even within diseased prostate or bladder). However we appreciate that this was in a small study population and our findings require corroboration in larger studies.

The reliability of measurements for different assessors is of vital importance for any potential device, so that consistent measurements are achievable for different users. The ICC of 0.73 (study 2) reveals that our device has good reliability, however, improving the reliability is likely to be achieved by adjusting for finger pressure, which was not done in this study. Despite this, the good ICC suggests that this method of elasticity assessment has potential for a future clinical application, in particular given the quantitative nature of assessment method, other techniques such as MRI for diagnosing the index tumour in prostate cancer currently achieve reliability of 0.57 [19]. The study showed consistent differences between different cadavers and between different measurement points on each tissue type. A quantitative model based only on the mechanical outcomes from the device was created using CART and revealed a sensitivity and specificity of 98% and 98% respectively on learning, which reduced to 77% and 70% on testing of this model with a validation cohort (study 2). The quantitative nature of the assessments enables such models to be created with high precision and this has potential clinical value as it removes the need for observer experience of pattern recognition such as exists with other elasticity technology like trans-rectal sonoelastography [20], [21].

The move into measuring elasticity in humans will bring with it a change in the environment within and surrounding the tissues of interest. In particular there will be more fluid in the tissues. This is likely to have an effect on the gross elasticity values that are obtained, however, because these structures are still distinct we feel that the significant differences which we detected will likely persist. Some evidence for this exists with prostate tissue observations which showed differing baseline elasticity values at different temperatures and if fixed or fresh. Interestingly, the differences between tumour and normal prostate were still evident in this study [22].

This study has a large translational element to it. The engineering achievement of creating a micro-scale device to assess tissue quality on such a small scale has potential clinical benefits in the future. Until recently, assessment of elasticity was carried out with large steel mechanical indenters with the tissue being assessed being placed between a baseplate and a mechanical indenter. The engineering challenge of miniaturising and “softening” the device for use in humans without compromising accuracy or reliability was until recently not possible. We plan further studies to allow us to transition into the clinical in-vivo environment during laparoscopy or robotic surgery, which we hope will provide assistance with tissue quality assessment that in turn will reduce the potential clinical consequences of incision into the prostate (PSM) or sphincter complex (incontinence) during surgery [23].

## Conclusion

This cadaveric study of tissue quality assessment around the prostate has demonstrated that DIP can determine clear consistent quantifiable differences in elasticity between bladder, prostate and sphincter complex. The quantitative nature of the assessment and consistency of results has clear potential advantages for clinical applications. Further work is being undertaken in the clinical environment to validate the cadaveric findings of this study and to deploy these in the minimally invasive environment.

## Supporting Information

Data S1 **Mechanical data for bladder and prostate.**
(XLS)

Data S2 **Mechanical data for prostate and sphincter.**
(XLS)
